# Transcranial Focused Ultrasound Neuromodulation: A Review of the Excitatory and Inhibitory Effects on Brain Activity in Human and Animals

**DOI:** 10.3389/fnhum.2021.749162

**Published:** 2021-09-28

**Authors:** Tingting Zhang, Na Pan, Yuping Wang, Chunyan Liu, Shimin Hu

**Affiliations:** ^1^Department of Neurology, Xuanwu Hospital, Capital Medical University, Beijing, China; ^2^Beijing Key Laboratory of Neuromodulation, Beijing, China; ^3^Center of Epilepsy, Institute of Sleep and Consciousness Disorders, Beijing Institute for Brain Disorders, Capital Medical University, Beijing, China

**Keywords:** transcranial focused ultrasound, neuromodulation, non-invasive brain stimulation, human, animal

## Abstract

Non-invasive neuromodulation technology is important for the treatment of brain diseases. The effects of focused ultrasound on neuronal activity have been investigated since the 1920s. Low intensity transcranial focused ultrasound (tFUS) can exert non-destructive mechanical pressure effects on cellular membranes and ion channels and has been shown to modulate the activity of peripheral nerves, spinal reflexes, the cortex, and even deep brain nuclei, such as the thalamus. It has obvious advantages in terms of security and spatial selectivity. This technology is considered to have broad application prospects in the treatment of neurodegenerative disorders and neuropsychiatric disorders. This review synthesizes animal and human research outcomes and offers an integrated description of the excitatory and inhibitory effects of tFUS in varying experimental and disease conditions.

## Introduction

Brain stimulation techniques have shown efficacy for ameliorating neurological and psychiatric disorders (Hoy and Fitzgerald, 2010). Invasive electrical brain stimulation modalities, such as vagus nerve stimulation (VNS) or deep brain stimulation (DBS), require the surgical placement of electrodes in the brain (Rahimpour et al., 2021; Wang Y. et al., 2021). The modulation effects of non-invasive strategies, such as transcranial direct current stimulation (tDCS) and repetitive transcranial magnetic stimulation (rTMS) are limited to the cortical surface (Hoy and Fitzgerald, 2010; Romanella et al., 2020). Transcranial focused ultrasound (tFUS) transmits low-intensity ultrasound into the brain non-invasively and focuses on deep brain regions. It is an emerging neuromodulation technology with the advantages of better spatial resolution and safety (Mehic et al., 2014).

The frequency of the ultrasound mechanical wave is higher than the range of human hearing (> 20 kHz). Ultrasound can be focused across solid structures and transmitted long distances with minimal power loss in soft biological tissues (Bystritsky et al., 2011). It has been the most widely used biomedical imaging modality for a long history. Ultrasound display different biological effect based on a different intensity. High-intensity (> 200 W/cm^2^) focused ultrasound induces permanent lesions through coagulation of cellular proteins and thermal ablation. It has been approved by the FDA for the treatment of tremors associated with Parkinson’s disease and essential tremors. Medium intensity (100–200 W/cm^2^) ultrasound has been used to break through the blood-brain barrier for drug delivery. Low-intensity (< 100 W/cm^2^) focused ultrasound is considered the main form of non-invasive neuromodulation (Kubanek, 2018).

Research on the potential clinical value of focused ultrasound as a neuromodulation method started half a century ago, with interest increasing dramatically over the past decade (Fini and Tyler, 2017). Studies using various parameters have shown that tFUS can modulate neural activity in the brains of animals and humans. It has the potential to modulate brain activities indicated by sensory or motor behaviors, functional magnetic resonance imaging (fMRI), electroencephalography (EEG), and near-infrared spectroscopy (NIRS) (Krishna et al., 2018; Legon et al., 2018b). The findings of *in vitro* and *in vivo* experiments have promoted the understanding of ultrasound neuromodulation. However, the high variability in experimental conditions, different ultrasound parameters, as well as partially conflicting results, led to contradictory interpretations. To achieve clinical application, it is important to clarify the excitatory and inhibitory effects of tFUS. In this review, we summarize the current findings of the brain modulatory effects of tFUS in both animal and human studies. We discussed findings of the excitatory or inhibitory effect of tFUS in light of varying experimental and disease conditions. Finally, the proposed mechanism for ultrasound neuromodulation was discussed.

## Literature Search

We searched the literature in the databases of the PubMed /MEDLINE/EMBASE with the following research string, in March 2021: (“Low-intensity focused ultrasound[Title/Abstract]” OR “Transcranial focused ultrasound[Title/Abstract]”) AND (“Neuromodulation[Title/Abstract]” OR “Brain Stimulation [Title/Abstract]”). Non-English language studies and duplicates were excluded. Studies were also excluded according to the following criteria: (1) Study investigating the diagnostic aspect of transcranial ultrasound rather than neuromodulation effects; (2) Study investigating the application of transcranial ultrasound in ablation neurosurgery or drug delivery; (3) Study focusing on the development of new ultrasound device; (4) Study published as the conference abstract without a full text, as dissertation or those published in books or only providing surrogate biomarkers. The resulting original articles that reported the use of tFUS in animals and human subjects to modulate brain activity were included (Tables 1, 2). Ultimately 41 studies were selected for discussion in this review, including 23 animals and 18 human studies.

**TABLE 1:** tFUS neuromodulation studies in animals.

References	Animal species	Targets	Sonication parameters	Key findings	Effect
Mohammadjavadi et al. (2019)	Normal mice	Midbrain	Focused, frequency = 500 kHz, PRF = 1.5 kHz, 8 kHz	tFUS-evoked motor responses independent of the peripheral auditory system; high correlation between tFUS pulse duration and electromyography response duration	Excitatory
Yuan et al. (2020)	Normal mice	Motor cortex	Unfocused, frequency = 500 kHz, PRF = 1 kHz, SD = 400 ms, DC = 40%, Isppa = 0.8 W/cm^2^	Ultrasound pulses induced motor response, neural activity, and rapid hemodynamic response selectively at the stimulation site	Excitatory
Kim et al. (2014)	Normal rats	Somatomotor area	Focused, frequency = 350 kHz and 650 Hz, DC = 30–100%, PRF = 0.1–2.8 kHz, SD = 150–400 ms, TBD = 0.25–5 ms	1–5 ms TBD, 50% DC, 300 ms SD stimulates the somatomotor area most effectively; 350 kHz frequency outperforms 650 kHz; pulsed outperforms the equivalent continuous sonication	Excitatory
Yu et al. (2016)	Normal rats	Right anterior cortex	Focused, frequency = 500 kHz, PRF = 2.0 kHz, Isppa = 0.74–4.6 mW/cm^2^	Low-intensity tFUS activated neurons correlated to intensity and SD. tFUS stimulation activated the focal site and propagating to surrounding areas over time	Excitatory
Moore et al. (2015)	Normal mice	Somatosensory barrel cortex	Focused, frequency = 350 kHz, DC = 42.8%, PRF = 2.0 kHz, TBD = 0.5 ms	Ultrasound stimulation induced depolarition of pyramidal cells	Excitatory
Fisher and Gumenchuk (2018)	Normal mice	Primary somatosensory cortex	Focused, frequency = 510 kHz, PRF = 1 kHz, Isppa = 0.69 or 3.5 W/cm^2^	Low-intensity FUS alters both the kinetics and spatial patterns of neural activity	Excitatory
Yoo et al. (2011)	Normal rats	Thalamus	Focused, frequency = 440–700 KHz, PRF = 100 Hz, TBD = 0.5 ms, DC = 50%, Isppa = 3.3–6 W/cm^2^	Ultrasound stimulation reduced the time to emergence from ketamine/xylazine anesthesia in rats	Excitatory
Baek et al. (2018)	Stroke mice	Lateral cerebellar nucleus	Focused, frequency = 350 kHz DC = 50%, PRF = 1 kHz, TBD = 0.5 ms, SD = 300 ms, Isppa = 2.54 W/cm^2^	tFUS enhanced the sensorimotor performance in mice of photothrombotic stroke	Excitatory
Lee et al. (2018)	Normal rats	Motor cortex	Focused, frequency = 600 kHz, PRF = 500 Hz, TBD = 1 ms, DC = 50%, Isppa = 8.8 W/cm^2^,14.9 W/cm^2^	Wearable miniature stimulator applied tFUS and elicited movements in both awake and anesthetized rats	Excitatory
Li et al. (2019)	Normal mice	Somatosensory cortex	Focused, frequency = 2 MHz, PRF = 1 kHz, DC = 30%, SD = 300 ms	Wearable ultrasound stimulator induced action potentials and evoked sonication−related movement behaviors	Excitatory
Darrow et al. (2019)	Normal rats	Thalamus	Focused,frequency = 3.2 MHz, PRF = 500 Hz, DC = 5–70%, Ispta = 0.01–36.03 W/cm^2^	tFUS suppressed a primary sensory pathway, much of the suppression could be attributed to thermal neuromodulation	Inhibitory
Dallapiazza et al. (2018)	Swine	Thalamus	Focused, Frequency = 1.145 MHz,650 kHz, 220 kHz, DC = 43.7%, Isppa = 25–30 W/cm^2^	Low intensity focused ultrasound inhibited sensory evoked potentials with a spatial resolution ∼2 mm.	Inhibitory
Min et al. (2011)	PTZ-injected epilepsy rats	Thalamus	Focused, frequency = 690 kH, TBD = 0.25–5 ms, PRF = 100 Hz, DC = 5%, Isppa = 130 mW/cm^2^	tFUS decreased epileptic EEG bursts and the epileptic behavior, did not cause any damage to the brain tissue	Inhibitory
Yang et al. (2020)	TLE mice	Hippocampus	Focused, frequency = 500 kHz, for modulation of the neural oscillation: PRF = 1 kHz, SD = 400 ms, DC = 40%; for seizure inhibition: frequency = 500 kHz, PRF = 500 Hz, SD = 30 s, and DC = 5%	The ultrasound modulated the neural oscillation and inhibit the seizure in TLE mice	Inhibitory
Chen et al. (2020)	PTZ-injected epilepsy rats	Cortex or hippocampus area	Focused, frequency = 0.5 MHz, various sonication parameters	Pulsed FUS exposure suppressed epileptic spikes and affected the PI3K-Akt-mTOR pathway	Inhibitory
Lin et al. (2020)	Macaques model of epilepsy, tissues from patients with TLE	Right frontal lobe	Focused, frequency = 750 kHz, Isppa = 2.02 W/cm^2^, PRF = 1 kHz, TBD = 0.3 ms, SD = 200 ms	tFUS reduces epileptiform activities and behavioral seizures in epileptic monkeys, inhibits the epileptiform discharges in neurons from patients, activates the inhibitory interneurons	Inhibitory
Kim et al. (2015)	Normal rats	Visual cortex	350 kHz, PRF = 100 Hz, Isppa = 1, 3, 5 W/cm^2^, DC = 1, 5, 8.3%	5%DC: VEPs magnitude decreased by tFUS with 3 W/cm^2^ Isppa, but not 1 W/cm^2^, slightly elevated when use 5 W/cm^2^ Isppa; 3 W/cm^2^ Isppa: VEPs magnitude decreased by tFUS with 5% DC, but not 1% DC, slightly elevated when use 8.3% DC	Inhibitory
Yoon et al. (2019)	Ovine	Primary sensorimotor area and its thalamic projection	Focused, frequency = 250 kHz; Excitatory effects: DC = 30, 50, 70, 100%, PRF = 100–1400 Hz, TBD = 0.5–3 ms, Isppa = 15.8, 18.2 W/cm^2^. Inhibitory effects: DC = 3, 5%, PRF = 30–100 Hz, TBD = 0.5, 1.0 ms, Isppa = 5.4, 11.6 W/cm^2^	tFUS with 70% DC showed superior stimulation efficiency and 0.5 ms TBD resulted in the highest response rate. The modulatory effects were transient and reversible. Repeated exposure to tFUS did not damage the brain tissue	Excitatory from EMG and inhibitory from SEPs
Verhagen et al. (2019)	Macaque	SMA, FPC	Focused, frequency = 250 kHz, PRF = 10 Hz, DC = 30%, PD = 30 ms, Isppa = 24.1 W/cm^2^ for SMA and 31.7 W/cm^2^ for FPC	tFUS changed each area’s connectional fingerprint, enhanced the connectivity activity in proximal areas, reduced coupling between the stimulated area and less closed regions. The effects were temporary and not associated with microstructural changes.	Change the connectional fingerprint
Folloni et al. (2019)	Macaque	Amygdala, ACC	Focused, frequency = 250 kHz, DC = 30%, Isppa = 64.9 W/cm^2^ in the amygdala and 18.8 W/cm^2^ in ACC	tFUS transiently and reversibly altered neural activity in subcortical and deep cortical areas with high spatial specificity; reduced the interconnection of the targeted areas with other regions.	Decreased network connectivity
Fouragnan et al. (2019)	Macaque	ACC	Focused, frequency = 250 kHz, PRF = 10 Hz, DC = 30%, PD = 30 ms	tFUS significantly changed ACC activity, altered strength of connectivity from ACC with 3 other regions, impaired translation of counterfactual choice values into actual behavioral change	Changed connectivity map
Wattiez et al. (2017)	Macaques	Visual cortex	Frequency = 320 kHz, Isppa = 5.6, 1.9 W/cm^2^	tFUS changed 40% of neurons in the recorded region, half showed a transient increase of activity and half showed decrease of activity.	Different neurons respond differently
Yang et al. (2021)	Macaque	Somatosensory areas 3a/3b	Focused, frequency = 250 kHz, DC = 50%, PRF = 2 kHz, Isppa = 2.0 W/cm^2^	tFUS suppressed tactile stimulus-evoked fMRI responses, directly activated neurons within the target at resting state	Neuron state-dependent effect

*ACC, anterior cingulate cortex; DC, duty cycle; FPC, frontal polar cortex; Isppa, the intensity of spatial-peak pulse average; PRF, pulse repetition frequency; SD, sonication duration; TBD, tone-burst duration; SMA, supplementary motor area; tFUS; TLE, temporal lobe epilepsy; PTZ, Pentylenetetrazol; fMRI, functional magnetic resonance imaging; VEPs, visual evoked potentials; EEG, electroencephalography; EMG, electromyography; SEPs, somatosensory evoked potentials.*

**TABLE 2:** tFUS neuromodulation studies in human.

References	Subjects/study design	Targets	Sonication parameters	indexes	Key findings	Modulate effects	Adverse effects
Lee et al. (2015)	Healthy volunteers (*n* = 1)/within- subjects, sham- controlled study	S1 (hand)	Focused, frequency = 250 kHz, PRF = 500 Hz, TBD = 1 ms, DC = 50%, SD = 300 ms, Isppa = 3 W/cm^2^	EEG, fMRI, tactile sensations task	tFUS elicited transient tactile sensations accurately to one finger, evoked cortical potentials similar to the SEPs generated by MN stimulation.	Excitatory	No
Lee et al. (2016b)	Healthy volunteers (*n* = 29)/within- subjects, single- blind, sham- controlled study	V1	Focused, frequency = 270 kHz, PRF = 500 Hz, PD = 1 ms, DC = 50%, SD = 300 ms, Isppa = 3 W/cm^2^	fMRI, EEG, phosphene perception task	tFUS elicited activation of V1 and other visual areas, elicited cortical evoked EEG potentials similar to classical VEPs	Excitatory	No
Lee et al. (2016a)	Healthy volunteers (*n* = 10)/within- subjects, double- blind, sham- controlled study	S1 and S2	Focused, frequency = 210 kHz, PRF = 500 Hz, PD = 1 ms, DC = 50%, SD = 500 ms, Isppa = 7.0–8.8 W/cm^2^	fMRI, EEG, tactile sensory task	tFUS targeting at S1 and S2 separately or simultaneously, elicited tactile sensations from the contralateral hand/arm areas	Excitatory	No
Liu et al. (2021)	Healthy subjects (*n* = 9)/sham-controlled, crossover study	S1	Focused, frequency = 500 kHz, SD = 500 ms, PRF = 300 Hz	64-channel EEG and ESI, sensory task	tFUS improved the subjects’ discrimination ability through excitatory effects	Excitatory	Not available
Gibson et al. (2018)	Healthy volunteers (*n* = 40)/between- subjects, single- blind, sham- controlled study	M1	Unfocused, Continuous, frequency = 2.32 MHz, Isppa = 34.96 W/cm^2^	TMS-induced MEPs	Ultrasound increased MEPs amplitude: 33.7% at 1 min, 32.2% at 6 min post stimulation)	Excitatory	No
Ai et al. (2018)	Healthy volunteers (*n* = 6)/Pre-post interventional study	Primary sensorimotor cortex (caudate area)	Focused, frequency = 500 kHz, PRF = 1 kHz, DC = 36% in 3T MRI experiment; frequency = 860 kHz, PRF = 0.5 kHz, DC = 50% in 7T MRI experiment; SD = 500 ms, Isppa: 6 W/cm^2^	fMRI scan (cortical BOLD at 3T and sub-cortical BOLD at 7T)	BOLD response was detected in the S1 in the 3T studies and in the caudate in the 7T study	Excitatory	Not available
Leo et al. (2016)	Healthy volunteers (*n* = 5)/within- subjects, sham- controlled study	M1	Focused, frequency = 500 kHz, PD = 0.36 ms; PRF = 1 kHz; DC = 36%; SD = 500 ms. Isppa: 16.95 W/cm^2^	fMRI scan during finger tapping task	tFUS induced BOLD response in the targeted cortical regions (in 3 of 6 subjects)	Excitatory	No
Yu et al. (2020)	Healthy subjects (*n* = 15)/within- subjects, sham- controlled study	S1 (leg area)	Focused, frequency = 500 kHz, SD = 500 ms, PRF = 300 and 3,000 Hz, Isppa = 5.90 W/cm^2^	64-channel EEG and ESI, EMP, voluntary foot tapping task	tFUS modulated MRCP source dynamics with high spatiotemporal resolutions; tFUS increased the MSPA; high ultrasound PRF enhances the MSPA outperforms low PRF.	Excitatory	Not available
Legon et al. (2014)	Healthy volunteers (*n* = 30 totally in 3 exp)/within- subjects, sham- controlled study	CP3	Focused, frequency = 500 kHz, SD = 500 ms; PRF = 1 kHz; DC = 36%, Isppa = 23.87 W/cm^2^	EEG activity recorded from four electrodes surrounding CP3 (C3, CP1, CP5 and P3); SEPs induced by MN stimulation; two-point discrimination tasks	tFUS spatially attenuated the amplitudes of SEPs, modulated the spectral content of sensory-evoked brain oscillations, enhanced the somatosensory discrimination abilities.	Inhibitory	No thermal or mechanical sensations
Mueller et al. (2014)	Healthy volunteers (*n* = 25)/within- subjects, sham- controlled study	CP3 and 1 cm laterally	Focused, frequency = 500 kHz, PD = 0.36 ms; PRF = 1 kHz, DC = 36%; SD = 500 ms, Isppa = 23.87 W/cm^2^	EEG data were acquired at sites C3, CP1, CP5, and P3; SEPs induced by MN stimulation	tFUS altered the phase distribution of intrinsic brain activity for beta frequencies, changed the phase rate of beta and gamma frequencies, affected phase distributions in the beta band of early SEPs	Inhibitory	Not available
					components.		
Legon et al. (2018a)	Healthy volunteers (*n* = 40)/within- subjects, sham- controlled study	Unilateral sensory nuclei of thalamus	Focused, frequency = 500 kHz; PD = 0.36 ms; PRF = 1 kHz; DC = 36%; Isppa: 7.03 W/cm^2^	EEG, SEPs induced by MN stimulation, two-point discrimination tasks	FUS inhibited the amplitude of the P14 SEPs, attenuated alpha and beta power, inhibited the locked gamma power, decreased the performance in tactile judgment task	Inhibitory	Not available
Legon et al. (2018b)	Healthy volunteers (*n* = 50)/within- subjects, sham- controlled study	M1	Focused, frequency = 500 kHz, PD = 0.36 ms; PRF = 1 kHz; DC = 36%; SD = 500 ms, Isppa = 17.12 W/cm^2^	Recruitment curves, MEPs, SICI, ICF, stimulus response reaction time task	tFUS inhibited the amplitude of MEPs, attenuated ICF, reduced reaction time in a motor task.	Inhibitory	Mild and moderate neck pain, sleepiness, muscle twitches, itchiness and headache.
Fomenko et al. (2020)	Healthy subjects (*n* = 16)/double-blinded study	M1	Annular ultrasound, frequency = 500 kHz, PRF = 1,000 Hz, SD = 0.1–0.5 s, DC = 10/30/50%, Isp ta = 0.93/2.78/4.63 W/cm^2^	TMS-induced resting peak-to-peak MEPs, visuomotor task	Ultrasound dose dependently suppressed TMS-elicited MEPs, increased GABAA- mediated SICI and decreased reaction time on visuomotor task	Inhibitory	No
Sanguinetti et al. (2020)	Healthy subjects (*n* = 50)/randomized, placebo-controlled, double-blind study	rIFG	Focused, frequency = 500 kHz, PD = 65 μs, PRF = 40 Hz, DC = 0.26%, SD = 30 s, Ispta = 130 mW/cm^2^	Mood questionnaires and EEG, fMRI and resting-state functional connectivity	30-s tFUS induced positive mood effects for up to 30 min, 2 min of tFUS modulated functional connectivity related to the rIFG and DMN	Unknown	Not available
Hameroff et al. (2013)	Chronic pain (*n* = 31)/double blind, sham- controlled, crossover study	Posterior frontal cortex, contralateral to the maximal pain	Unfocused, Continuous, frequency = 8 MHz, MI = 0.7, max Intensity = 152 mW/cm^2^	Heart rate, systolic and diastolic blood pressure, oxygen saturation, numerical rating scale for pain, Visual Analog Mood Scale	15 s ultrasound significantly improved mood at 10 and 40 min following stimulation	Excitatory	Transient headache exacerbation following stimulation (1 subj)
Monti et al. (2016)	Post-traumatic disorder of consciousness 19 days post-injury (*n* = 1)/Case report, part of an ongoing clinical trial	Thalamus	Focused frequency = 650 kHz, PD = 0.5 ms; DC = 5%, PRF = 100 Hz. Ispta = 720 mW/cm^2^.	Chart review, response to command, and reliable communication (by yes/no head gesturing)	3 days of ultrasound treatment the patient demonstrated emergence from minimally conscious state. 5 days after treatment, the patient attempted to walk.	Excitatory	No
Beisteiner et al. (2020)	AD patients (*n* = 35)/multicenter pre-post study	AD relevant brain areas and the global brain	Single ultrashort (3 μs) ultrasound pulses, typical energy levels = 0.2–0.3 mJ/mm^2^, PRF = 1–5 Hz, maximum energy flux density = 0.25 Mj/mm^2^ at 4 Hz, maximum Ispta = 0.1 W/cm^2^, maximum number of pulses per treatment = 6,000	EEG data recorded at CP3, SEPs, neuropsychological tests, MRI	TPS treatment significantly improved neuropsychological scores, the effects last up to 3 months and correlates with an upregulation of the memory network	Excitatory	No
Badran et al. (2020)	Healthy subjects (*n* = 19)/double-blind, sham-controlled, crossover study	Right anterior thalamus	Focused, frequency = 650 kHz, PD = 5 ms, PRF = 10 Hz, DC = 5%, SD = 30 s, Ispta = 719 and 995 W/cm^2^	Sensory threshold, sensory, pain, and tolerance thresholds to a thermal stimulus	Thermal pain sensitivity was significantly attenuated after tFUS treatment	Inhibitory	No

*AD, Alzheimer’s disease; BOLD, blood oxygenation level dependent; DC, duty cycle; DMN, default mode network; EMG, electromyography; ESI, electrophysiological source imaging; Ispta, the intensity of spatial-peak temporal average; M1, primary motor cortex; MEPs, motor-evoked potential; MRCP, movement-related cortical potential; MSPA, MRCP source profile amplitude; MN, median nerve; PRF, pulse repetition frequency; rIFG, right inferior frontal gyrus; S1, left primary and secondary somatosensory cortex; S2, left secondary somatosensory cortex; SEPs, somatosensory evoked potentials; SD, sonication duration; TBD, tone-burst duration; TPS, transcranial pulse stimulation; SICI, short interval intracortical inhibition; TMS, transcranial magnetic stimulation; V1, primary visual cortex; VEPs, visual evoked potentials.*

## Main Ultrasound Parameters

The neuromodulation effect of ultrasound depends on the stimulation parameters used. Pulsed ultrasound is the main form of neuromodulation application to minimize the probability of tissue heating or damage. Fundamental frequency, pulse repetition frequency (PRF), duty cycle (DC), sonication duration (SD), and intensity are the five most important elements that define a sonication protocol. The fundamental frequency refers to the number of oscillatory cycles per unit time and is inversely proportional to wavelength. Fundamental frequency significantly affects the spatial targeting of brain regions. A higher fundamental frequency induces stimulation with a tighter focus (when the frequency is > 1 MHz, the diameter can be as narrow as few millimeters). But ultrasound with higher fundamental frequency has more transcranial attenuation and scattering. 200–650 kHz have been used in most human and animal studies. PRF refers to the rate of the pulses delivered. DC is the proportion of each pulse filled with ultrasound cycles, which is an important parameter that determines the direction of the neuromodulation effect. SD refers to the total time from the onset of the first pulse to the termination of the last pulse, which may determine the total intensity and tissue heating. The combination of PRF, DC, and SD may affect the inhibition or excitation of cortical neurons. The intensity of acoustic exposure is commonly characterized by two parameters: the spatial-peak temporal average (I_spta_) and the spatial-peak pulse average (I_sppa_). I_spta_ measures the average intensity during the entire sonication therapy and is proportional to SD. I_sppa_ measures the average intensity of a single pulse and provides estimates of short-term mechanical biological effects. The mechanical index (MI) is an indicator of the risk of potentially destructive biomechanical effects on tissues, such as inertial cavitation. It is equal to the peak negative pressure divided by the square of the fundamental frequency. Considering that thermal and mechanical effects may cause brain damage, FDA guidelines for cephalic ultrasound suggest a maximum safety value range of Ispta < 94 mW/cm^2^, Isppa < 190 W/cm^2^, and MI < 1.9 to avoid cavitation and heating in humans (United States Food and Drug Administration. Marketing clearance of diagnostic ultrasound systems and transducers. Draft guidance for industry and food and drug administration staff. 2019).

## Neuromodulation Effects in Animals

To use ultrasound for neuromodulation therapy, an important issue is to clarify its excitatory or inhibitory effects on the central nervous system. We reviewed the reports of the neuromodulatory effects of ultrasound in animals. Studies have been conducted in mice, rats, ovines, swine, sheep, and macaques with different ultrasound parameters, and brain targets. The reported brain targets included the motor cortex, midbrain, somatosensory cortex, hypothalamus, right anterior cortex, hippocampus, thalamus, and visual cortex. The excitatory and inhibitory effects vary with the stimulation targets and sonication parameters (Table 1).

## Excitatory Effect

Several studies have reported excitatory effects of tFUS targeting the cortex and deep brain areas. For targeting at the motor cortex, unfocused ultrasound with a frequency of 500 kHz evokes motor responses, and the responses are not related to the stimulation of the peripheral auditory system, which emphasizes the direct action of motor neurons in the brain (Mohammadjavadi et al., 2019). Another study used low-intensity tFUS (500 kHz) to stimulate mouse motor cortical regions and demonstrated that tFUS induced tail movement, neural activity, and hemodynamic responses (cortical blood flow, CBF) immediately and returned to baseline at ∼5.5 s. The relationships between CBF and key ultrasound parameters showed that the CBF responses increasing with increasing intensities (Isppa = 0.2–1.1 W/cm^2^, SD = 400 ms, DC = 40%) and SD (SD = 50–400 ms, Isppa = 0.8 W/cm^2^, DC = 40%), while had a weak dependence on DC (DC = 10–40%, SD = 50–400 ms, Isppa = 0.8 W/cm^2^) (Yuan et al., 2020). Kim et al. (2014) examined the stimulation effects of tFUS with a range of sonication parameters administrated to the somatomotor cortex of rats *in vivo*. Based on comparison experiments, the authors found that 50% of DC outperforms 30 and 70%. Operating at 50% DC, the use of TBDs in the range of 1–5 ms, serving as an effective pulsing scheme, 350 kHz fundamental frequency outperforms 650 kHz, pulsed tFUS outperforms equivalent continuous sonication stimulate the target cortex most effectively. Further, for the parameter of SD, they found that 400 ms required higher Isppa for eliciting motor activity than 300 ms, which may indicate that the longer SD might have recruited inhibitory neural circuits or neural cells. Overall, this study found non-linear neural responses to tFUS that might include the activation of motor neurons and inhibitory interneurons (Kim et al., 2014).

For other cortex regions, Yu et al. (2016) demonstrated that low intensity (Ispta < 1 Mw/cm^2^) 500 kHz pulsed tFUS targeting the right anterior cortex of rats evoked time-locked activation of the brain, as correlated to intensity and SD. They also suggested electrophysiological source imaging as a useful tool to quantify tFUS effects, guide its use for neuromodulation (Yu et al., 2016). The study of Moore et al. (2015) recorded local field potential fluctuations in the motor cortex in response to ultrasound stimulation of the mouse somatosensory barrel cortex. Ultrasound stimulation at 350 kHz and 42.8% DC induced depolarization of cerebral cortical pyramidal neurons, which indicates an excitatory effect of nerve transmission (Moore et al., 2015). Fisher and Gumenchuk (2018) demonstrated that tFUS with a frequency of 510 kHz reduced the latency and concentrates the spatial patterns of neural activity in the primary somatosensory cortex. The findings suggest the using of tFUS to target and alter spatial aspects of sensory receptive fields on the cerebral cortex (Fisher and Gumenchuk, 2018). As for deep brain targets, the research of Yoo et al. (2011) revealed that pulsed FUS targeting at the thalamus significantly decreased the time to emergence from ketamine/xylazine anesthesia in rats. This study provided early evidence for the excitatory neuromodulatory potential of tFUS targeting the deep brain area (Yoo et al., 2011). The study of Baek et al. (2018) provides the first evidence in the disease model showing that tFUS-induced cerebellar modulation improved impaired sensorimotor function in stroke mice and could be a potential strategy for post stroke recovery. They hypothesized that the effectiveness could be attributable to long-term potentiation of hypoactive neural connections between the motor cortex and deep cerebellar nucleus induced by tFUS. Longer follow-up studies are necessary to fully confirm the effects of tFUS on post stroke recovery (Baek et al., 2018).

## Inhibitory Effect

Three studies targeting the thalamus showed inhibitory effects of tFUS. The study of Darrow et al. (2019) revealed that 3.2 MHz tFUS targeting the rat thalamus reversibly suppressed somatosensory evoked potentials (SEPs) in a spatially and intensity-dependent manner. The effect was independent of the parameters of DC, peak pressure, or modulation frequency. The ultrasound frequency used in this study is higher than most other studies, which may cause an obvious thermal effect and lead to the suppression effect (Darrow et al., 2019). The study of Dallapiazza et al. (2018) demonstrated that low-intensity focused ultrasound with frequencies of 1.1 MHz, 220 kHz, and 650 kHz inhibited sensory evoked potentials with a spatial resolution of ∼2 mm in swine, supporting its potential use in non-invasive brain mapping. Longer ultrasound pulses delivered over prolonged periods tend to result in a more substantial and sustained decrease in neural function. The physiological effects lasted for a period of several minutes without inducing tissue heating or histological damage (Dallapiazza et al., 2018). Kim et al. (2015) reported that the application of pulsed 350 kHz tFUS using a 5% DC and Isppa intensity of 3 W/cm^2^ to the visual cortex area suppressed the magnitude of visual evoked potentials (VEPs) in rats. Under the same conditions, higher intensity (5 W/cm^2^) or DC (8.3%) induced slight elevation in VEPs (Kim et al., 2015). Yoon et al. (2019) investigated the bimodal effects of tFUS in the modulation of the sensorimotor cortex and thalamus in an ovine model by evaluating the rate and magnitude of electrophysiological responses to a wide range of sonication parameters. The study suggests that a shorter SD (≤ ∼500 ms) at a higher DC (30%) favored excitation, and a longer SD (∼1 min) at a lower DC (≤ 10%) resulted in suppression. For excitation effects, the use of 15.8 W/cm^2^ Isppa generated a higher response rate than the use of 18.2 W/cm^2^. This study provided important evidence for the effects of varying sonication parameters on neuromodulation response (Yoon et al., 2019).

The above studies that reported excitatory and inhibitory regulation effects demonstrated the feasibility of tFUS to the clinical application of neural modulation. However, the ultrasound parameters used by different research groups are different, no two studies used consistent parameters. The rules of the neuromodulation effect corresponding to parameter changes are still unclear. Change of any one of the main ultrasound parameters may produce a change of efficacy of excitatory or inhibitory effects, with the correlation relationship is considered to be non-linear. Nevertheless, the influence of any parameters on the modulatory direction is inconclusive, and the combination of multiple parameters seems more difficult. Further research should be conducted to explore how to precisely control parameters to achieve a specific adjustment purpose.

Other evidence of inhibitory neuromodulation effects comes from research in animal models of epilepsy. An early study by Min et al. (2011) reported that low-intensity, pulsed FUS (690 kHz) sonication suppressed the number of epileptic signal bursts and severe epileptic behavior using an acute epilepsy model in animals. Yang et al. (2020) delivered pulsed closed-loop transcranial ultrasound stimulation with a frequency of 500 kHz to the hippocampus to modulate neural oscillation and effectively inhibited the seizure of a temporal lobe epilepsy (TLE) mouse. The study of Chen et al. (2020) reported that pulsed tFUS (500 kHz) effectively suppressed epileptic spikes in an acute epilepsy animal model and found that ultrasound pulsation interferes with neuronal activity. Zhengrong Lin et al. (2020) demonstrated that low-intensity pulsed FUS could improve the electrophysiological activities and behavioral outcomes in non-human primate models of epilepsy. The study also demonstrated that ultrasound suppressed abnormal epileptiform activities of neurons from human epileptic slices (Lin et al., 2020). The suppression of epileptic neuro-electric activity and the behavior of tFUS may mainly indicate inhibitory effects.

Moreover, most current animal experiments are performed using anesthetized animal models to prevent animal motion during ultrasound administration. A recently published study by Wang X. et al. (2021) demonstrated that the behavioral states, especially anesthesia, modulate the ultrasound stimulation-induced neural activity. The effect of pharmacological sedation on tFUS induced effects is not clear and may obfuscate the interpretation of the tFUS neuromodulation response (Jerusalem et al., 2019). Studies in awake and freely moving animals are needed. Lee et al. (2018) conducted the first study that using awake rats to evaluate the neuromodulatory efficacy of tFUS. They developed a miniature tFUS headgear with a frequency of 600 kHz, and the stimulation of motor cortical areas by their configuration elicited body movements from various areas in freely moving rats. Compared with the anesthetic rats, the stimulation in awake rats induced an increased response rate with reduced variability and shorter latency. However, the lack of measurement of electrophysiological signals in this study limited its information (Lee et al., 2018). Li et al. (2019) designed a miniature and lightweight head-mounted ultrasound stimulator that can be used in freely moving mice. When target at the primary somatosensory cortex barrel field, the device evoked head-turning behaviors and action potentials recorded *in situ* (Li et al., 2019). The application of stimulation in awake animals is valuable for the research on the neuromodulation effects and mechanisms of tFUS, especially for investigations that are not possible with anesthesia, such as social behavioral studies and disease models that are influenced by anesthesia (e.g., epilepsy).

## Neuromodulation in Human Subjects

The excitatory effects of tFUS were identified in healthy human subjects in 9 studies as indicated by fMRI and EEG. Six studies showed the inhibitory effects of TUS indicated by SEPs, motor induced potentials (MEPs), and intracortical facilitation (ICF). The modulated brain targets included the motor cortex, somatosensory cortex, thalamus, caudate nuclei, and visual cortex. Several studies have reported the effect of FUS stimulation on patients with chronic pain, posttraumatic disorder of consciousness, and Alzheimer’s disease (AD) (Table 2).

## Excitatory Modulation

Studies provide evidence that FUS stimulation can activate the brain to produce sensory and motor nerve responses without any external stimulation. Three studies conducted by Lee et al. (2015) showed excitatory effects of tFUS on the human primary and secondary somatosensory cortex and visual cortex. tFUS targeting the human hand somatosensory cortex (S1) elicited somatosensory sensations with anatomical specificity up to a finger and evoked EEG potentials similar to the classical SEPs generated by median nerve stimulation (Lee et al., 2015). In another study, they reported that FUS targeting to the human visual cortex (V1) induced the perception of phosphine and activated a network of regions involved in visual and higher-order cognitive processes (Lee et al., 2016b). They also reported that tFUS application to the human bilateral primary somatosensory cortex (S1) and secondary somatosensory cortex (S2) elicited various tactile sensations in the targeted hand area. This study also showed the possibility of simultaneous stimulation of the S1 and S2 (proximal to each other) with ultrasound, which has not been feasible with conventional non-invasive brain stimulation approaches such as TMS or tDCS. The ability to selectively stimulate multiple human brain areas in a spatially restricted manner may offer an unprecedented opportunity to study the causal relationships between brain activity and subsequent efferent behaviors (Lee et al., 2016a). Ai et al. (2018) combined tFUS with high-field 7T fMRI in humans and evaluated its neuromodulation effect by the BOLD response. tFUS stimulation targeted individual finger representations within M1 increased the activation volumes of the M1 thumb representation in a spatially restricted manner. These results provide a more detailed perspective on the spatial resolution of tFUS for neuromodulation of individual finger representations within a single gyrus reflected by the BOLD response (Leo et al., 2016; Ai et al., 2018).

FUS stimulation modulates the neural response induced by behavioral tasks and TMS-induced MEPs. Liu et al. (2021) evaluated the interference of tFUS stimulation at S1 on the performances in a mechanical vibration frequency discrimination task in a group of healthy human participants. The behavioral results indicated that low-intensity tFUS stimulation improved vibration frequency discrimination capability. EEG and electrophysiological source imaging (ESI) results revealed that tFUS improved sensory discrimination capability through exciting the targeted sensory cortex (Liu et al., 2021). The study by Gibson et al. (2018) used a diagnostic imaging ultrasound system to stimulate the motor cortex. Increased TMS-induced MEPs amplitude (34%) was recorded up to 6 min after stimulation but disappeared 11 min later (Gibson et al., 2018). This result contrasts with the research of Legon et al., who reported tFUS inhibited MEPs induced by TMS (Legon et al., 2018b). As discussed by the authors, the different findings may be caused by stimulation parameters and other methodological factors. Yu et al. (2020) demonstrated the neuromodulatory effects of low-intensity tFUS on human voluntary movement-related cortical potential (MRCP). Through ESI, the results showed that tFUS modulates MRCP source dynamics with high spatiotemporal resolutions and significantly increases the MRCP source profile amplitude (MSPA), and further, a high PRF enhances the MSPA outperforms a low UPRF does. This study provides the first evidence that tFUS enhances human endogenous motor cortical activities through excitatory modulation (Yu et al., 2020).

## Inhibitory Modulation

Five studies by one research group reported the inhibitory modulation effect of tFUS targeting the primary somatosensory cortex (CP3), unilateral sensory nuclei of the thalamus, and primary motor cortex. They used similar ultrasound parameters with the 500 kHz frequency, 36% DC, 500 ms SD, and 1 kHz PRF. The first study reported that tFUS targeting CP3 significantly attenuated the amplitudes of SEPs and modulated the spectral content of brain oscillations elicited by median nerve stimulation, while enhanced the somatosensory discrimination abilities of participants. Importantly, this research also showed that the influence of tFUS on brain activity can be spatially restricted within 1 cm (Legon et al., 2014). The following study by Mueller et al. (2014) found that tFUS preferentially affected the phase distribution of the beta band and modulated the phase rate of both beta and gamma frequencies. The third study reported that tFUS targeting the thalamus attenuated the SEPs generated in the ventral-lateral nucleus and serially connected cortical regions. The study provided initial evidence that tFUS can non-invasively modulate subcortical areas of the human brain with good spatial precision and resolution (Legon et al., 2018a). The fourth study showed that tFUS inhibits the amplitude of single-pulse MEPs and attenuates intracortical facilitation, which confirms previous results that ultrasound results in effective neuronal inhibition. tFUS did not affect short interval intracortical inhibition (SICI) or reduce reaction time in a simple stimulus response task in this study (Legon et al., 2018b). The studies of Fomenko et al. (2020) reported that tFUS targeting at the motor cortex displayed inhibitory effects on cortical excitability, but the effects on SICI were inconsistent. Furthermore, other studies have reported heterogeneous effects of FUS targeting on the motor cortex, demonstrating increased cortical excitability or the amplitude of MEPs after prolonged sonication (Gibson et al., 2018; Yu et al., 2020).

Like animal studies, human studies have inconsistent conclusions about whether ultrasound modulation is exciting or inhibiting. In addition to the influence of sonication parameters, differences in detection methods also may introduce inherent variability. For example, the study of Leo et al. (2016) and Legon et al. (2018a) used similar FUS protocols (500 kHz frequency, 36% DC, and 1 kHz RPF) targeting the M1 cortex demonstrated excitatory or inhibitory effect, respectively. Leo et al. (2016) reported BOLD fMRI signals were induced by tFUS, while Legon et al. (2018a) detected reduced amplitude of MEPs and intracortical facilitation. Further prospective studies are needed to elucidate region- and neuron-specific sensitivity to focused ultrasound with a wider range of parameters.

## Clinical Application

Preliminary research has explored the role of this technology in the treatment of human brain functional diseases. In healthy subjects, Sanguinetti et al. (2020) reported two experiments that demonstrated that tFUS targeting the right inferior frontal gyrus (rIFG) enhances mood and changed the functional connectivity in networks related to emotional regulation. These results suggest that tFUS may be useful in modulating mood and emotional regulation networks (Sanguinetti et al., 2020). Badran BW conducted a controlled, double-blind study to investigate whether sonication targeting deep brain structures produces quantifiable antinociceptive effects in healthy adults. The study reveals that two 10-min sessions of tFUS delivered to the right anterior thalamus produced significant antinociceptive effects in thermal pain threshold ratings, suggesting that tFUS appears to be able to focally target deep brain structures and modulate pain perceptions (Badran et al., 2020).

Four studies have provided evidence for its potential in disease treatment in patients with chronic pain, a minimally conscious state, AD and epilepsy. Hameroff et al. (2013) reported a double-blind, sham-controlled crossover study applying 8 MHz unfocused transcranial ultrasound stimulation targeted to the posterior frontal cortex in 31 patients with chronic pain. They found improvement in subjective mood 10 and 40 min after stimulation, suggesting that ultrasound treatment can beneficially affect mental state. However, the study was limited in clinic time and unable to perform extensive psychological testing (Hameroff et al., 2013). Monti et al. (2016) published a clinical case report showed that thalamic tFUS stimulation improved the minimally conscious state in a patient after acute brain injury. An important study by Beisteiner et al. (2020) introduces a clinical sonication technique based on single ultrashort ultrasound pulses (3 μs) repeated every 200–300 ms transcranial pulse stimulation (TPS), which markedly differs from others. The TPS was applied to brain regions of patients with probable AD. The researchers found that the treatment significantly improved neuropsychological scores and upregulated corresponding memory network. This study provided comprehensive preclinical and clinical feasibility, safety, and efficacy data for TPS in the treatment of AD. Thus, widespread neuroscientific application and translation of the method to clinical therapy is encouraged (Beisteiner et al., 2020). An early research by Brinker et al. (2020) developed a laboratory-built experimental device platform and successfully delivered repetitive low-intensity tFUS across the hippocampus in seizure onset zones of patients with drug-resistant TLE. Further study is still ongoing for investigating the effects of tFUS therapeutic neuromodulation in the patients with TLE (Brinker et al., 2020). Overall, to move tFUS forward as a potential therapy for brain diseases, more researches in patients are needed to explore targeting, dosing, and parameter optimization modes.

## Evidence for Mechanisms

The precise mechanisms of neuromodulation with ultrasound are unclear. Studies have explored the mechanism for the neuromodulation effect of tFUS from the perspective of brain networks, neural cells, and molecules (Table 1). The effect of ultrasound neuromodulation on brain network connections has been reported in researches using macaques and rats. Verhagen et al. (2019) reported that 40 s of ultrasound stimulation in macaques modulated the brain activation for more than 1 h and the ultrasound displays offline and sustained impact on the connectional fingerprint of stimulated brain regions. The study of Folloni et al. (2019) demonstrated that a tFUS protocol impacts activity in the subcortical brain structure of the amygdala and deep cortical region of the anterior cingulate cortex in macaques. The stimulation suppressed the connectivity of the targeted brain area to its network (Folloni et al., 2019). The study of Fouragnan et al. (2019) investigated how representations of counterfactual choices are held in memory and guide behavior in macaque monkeys. tFUS was used to focally alter the neural activity to examine the causal importance of the anterior cingulate cortex (ACC) for behavior. They found that tFUS significantly impaired translation of counterfactual choice values into actual behavioral change and changed the activity and connectivity maps of the ACC (Fouragnan et al., 2019). The study of Zhang et al. (2021) reported that FUS reduced the network connections of epilepsy circuits and change the structure of the brain network at the whole-brain level. The adjustment effect of tFUS on neural network connection may be the mechanism of its repeated application to improve brain diseases.

At the cellular level of neurons, two studies reported bimodal neural modulation effects of tFUS in macaques. The study of Wattiez et al. (2017) assessed the neuromodulatory effects of tFUS in awake behaving monkeys by recording discharge activity from a brain region (supplementary eye field) reciprocally connected with the stimulated region (frontal eye field). They demonstrated that stimulation in the visual cortex significantly changed the activity of 40% of neurons in the recorded region. Half of the neurons showed transiently increased activity, and the other half showed decreased activity. In this study, the ultrasonic effect was quantified based on the direct measurement of the intensity of the modulation induced on a single neuron in a freely performing animal. In particular, the study suggests that different neurons respond differently to tFUS stimuli and indicates further parametric studies should pay attention to the regulation of neural activity at the cell level (Wattiez et al., 2017). Yang et al. (2021) reported that medium amplitude tFUS (425 kPa free-field at 250 kHz) in the macaque brain modulated the activity of neurons at the target in dual directions (excitation and suppression). This simultaneous excitatory and suppressive neuromodulation may be mediated by activation of large excitatory pyramidal and small inhibitory interneurons, respectively. This study first examined the neuromodulation effects of FUS at the whole-brain level and the ability of concurrent FUS and MRI to evaluate causal interactions between functional circuits and neuron-class selectivity (Yang et al., 2021).

Several studies provided evidence for the mechanism of the neuromodulation effect at the molecular level. Recent investigations on the interactions between sound pressure waves and brain tissue suggest that ultrasound primarily exerts its modulatory effects through mechanical action on cell membranes, notably affecting ion channel gating (Kubanek et al., 2016). Liang et al. (2020) explored the efficacy and mechanisms of tFUS in treating pain caused by soft tissue injury. Low-intensity focused ultrasound relieved pain, and the mechanism could be attributed to decreasing the release of analgesic substances from the nerve and reducing local inflammatory factors (Liang et al., 2020). Na Pang et al. (2021) demonstrated that transcranial ultrasound stimulation was effective in modulating the learning behaviors of mice and the expression of apoptosis, oxidative stress, and inflammation. Xu et al. (2020) demonstrated that low-intensity ultrasound is able to stimulate DA release and helps to regenerate dopaminergic neurons in a mouse model of Parkinson’s disease (PD). Huang et al. (2019) reported that non-invasive low-intensity pulsed ultrasound stimulation improved c-fos expression and reduced the incidence rate (*p* < 0.05) and length of primary cilia (*p* < 0.01) of neurons in the rat CA1 hippocampus. Bobola et al. (2020) reported that acutely applied tFUS activated microglia to colocalize with Aβ plaques in a mouse model of AD, and 5 days of tFUS cleared almost 50% of the Aβ plaque. The study of Chen et al. (2020) suggested that pulsed tFUS exposure effectively suppresses epileptic spikes in an acute epilepsy animal model and revealed that ultrasound pulsation affects the PI3K-Akt-mTOR pathway, which might be the molecular mechanism. These studies only provide preliminary clues, and more studies are needed in the future.

## Safety of Transcranial Focused Ultrasound

Currently, ultrasound for neuromodulation in humans generally follows the guidelines of the FDA for adult cephalic applications. Legon et al. (2020) qualitatively evaluated minor adverse events in seven independent experiments using different ultrasound protocols for human neuromodulation. The intensity (Isppa) used in these studies ranged from 11.56 to 17.12 W/cm^2^ that is considerably lower than FDA thresholds for ultrasound diagnostics. The parameter of Ispta, which is defined as the Isppa multiplied by the duty factor, in these studies was above FDA thresholds for diagnostics. They found that none of the participants experienced serious adverse effects, and 7/64 reported mild to moderate symptoms probably related to ultrasound treatment. The symptoms include neck pain, attention problems, muscle twitches, and anxiety. They concluded that the symptom rate and type induced by tFUS are similar to other forms of human non-invasive neuromodulation, such as TMS and tDCS, these two have been used as safe forms of human neuromodulation. Despite the causation role of ultrasound has not been defined, the findings suggest limiting the intensity used in future ultrasound experiments for neuromodulation (Legon et al., 2020). Gaur et al. (2020) examined a range of experimental parameters, including the number of focal spot locations, the number of FUS bursts applied to each spot, the timing between FUS sessions, and applied acoustic intensity, to investigate the safety of FUS neuromodulation. The *in situ* intensities were 9.5 W/cm^2^ in macaques and 6.7 W/cm^2^ in sheep, similar to and slightly higher than previously reported Ispta values of up to 4.4 W/cm^2^ in humans. Repeated FUS neuromodulation at various intensity levels for multiple days did not cause histologic damage. The study suggesting that the neuromodulation protocols evaluated do not cause tissue damage and provide important information for the safety profiles of FUS neuromodulation (Gaur et al., 2020).

## Discussion

Overall, tFUS is a promising non-invasive brain stimulation technology. The current findings in both animal and human studies reported both the excitatory and inhibitory modulatory effects of tFUS on brain activity. These findings confirmed the application prospects of this technology in the treatment of brain functional diseases. However, the current research does not clarify the correlation among the roles of the stimulus parameters in excitatory or inhibitory modulatory effects. The excitatory and inhibitory effects shown in current research are mostly on a macro level, and it is not clear whether these effects are derived from inhibitory or excitatory neuronal activity. Inhibitory effects may come from the excitement of inhibitory neurons or the inhibition of excitatory neurons, and the opposite is also possible. The physiological state (active or resting) of neurons in the stimulated area and its connected areas and how these neurons interact during the modulation process could also influence the results. Like clinical trials, animal experiments have been conducted in normal animals and disease model animals, the targets include the sensory cortex, motor cortex, and deep nuclei such as the thalamus. The range of ultrasonic parameters used in animal studies were similar to that in clinical trials. Due to the differences in skull size and geometry of the brain, the parameters from tFUS used in animals are less likely to be translated to humans. Further animal studies should focus on the change law of excitation or inhibition effect with the fine change of parameters, and to further reveal the mechanism of regulation effect at the nerve conduction, cellular and molecular levels. The safety profile, effectiveness, and finer device parameters in humans in either healthy or diseased conditions need further research.

## Author Contributions

TZ drafted this report. SH, CL, NP, and YW revised and expanded. All authors contributed to the article and approved the submitted version.

## Conflict of Interest

The authors declare that the research was conducted in the absence of any commercial or financial relationships that could be construed as a potential conflict of interest.

## Publisher’s Note

All claims expressed in this article are solely those of the authors and do not necessarily represent those of their affiliated organizations, or those of the publisher, the editors and the reviewers. Any product that may be evaluated in this article, or claim that may be made by its manufacturer, is not guaranteed or endorsed by the publisher.
